# Surfaces away from horizons are not thermodynamic

**DOI:** 10.1038/s41467-018-05433-9

**Published:** 2018-07-30

**Authors:** Zhi-Wei Wang, Samuel L. Braunstein

**Affiliations:** 1 College of Physics, Jilin University, Changchun, 130012 People’s Republic of China; 2 Computer Science, University of York, York, YO10 5GH UK

## Abstract

Since the 1970s, it has been known that black-hole (and other) horizons are truly thermodynamic. More generally, surfaces which are not horizons have also been conjectured to behave thermodynamically. Initially, for surfaces microscopically expanded from a horizon to so-called stretched horizons, and more recently, for more general ordinary surfaces in the emergent gravity program. To test these conjectures we ask whether such surfaces satisfy an analogue to the first law of thermodynamics (as do horizons). For static asymptotically flat spacetimes we find that such a first law holds on horizons. We prove that this law remains an excellent approximation for stretched horizons, but counter-intuitively this result illustrates the insufficiency of the laws of black-hole mechanics alone from implying truly thermodynamic behavior. For surfaces away from horizons in the emergent gravity program the first law fails (except for spherically symmetric scenarios), thus undermining the key thermodynamic assumption of this program.

## Abstract (web summary)

It has been conjectured that gravity may emerge from an entropic force arising on a holographic screen due to its purportedly intrinsic thermodynamic properties. Here, the authors test this conjecture by demonstrating that this key assumption of entropic force is inconsistent with general relativity.

## Introduction

In 1973, Bardeen et al.^1^ derived the laws of black-hole mechanics which are in direct analogy with the laws of thermodynamics. Together with the discovery of Hawking radiation^2^, the truly thermodynamic behavior of black-hole horizons became well established. Indeed such thermodynamic behavior is now well accepted for all spacetime horizons, including those due to accelerated observers^3,4^ and cosmological horizons^5^.

Later, other surfaces were also attributed with thermodynamic properties. Firstly, stretched horizons were claimed to be thermodynamic, effectively acting as radiating black bodies^6^ with a temperature *T* = *κ*/(2*π*) determined by their local surface gravity *κ* and an entropy (a “state variable”) associated with a statistical mechanical interpretation of black-hole entropy^6,7^. An explicit rederivation of the laws of black-hole mechanics has not been previously carried out for stretched horizons. More recently, a class of ordinary surfaces has been conjectured to behave thermodynamically, forming the key assumption in the emergent gravity program^8^. This thermodynamic attribution was justified in part by using it in a heuristic derivation of the full Einstein field equations in static asymptotically flat spacetime^8^.

Here, we ask whether canonical general relativity is consistent with the assumption that such ordinary surfaces can be rigorously seen to behave thermodynamically. We attack this question by focusing on the analogue to the first law of thermodynamics. Originally, this law was derived in an analysis that was specialized to the behavior of horizons^1^. We remove this specialization to reveal the behavior of ordinary surfaces in an analysis of the first law. Here, we report that the first law holds to an excellent approximation for stretched horizons. Finally, with the exception of fully spherically symmetric scenarios, we find that for ordinary surfaces in the emergent gravity program that the first law fails to hold.

## Results

### Energy conservation

**Fig. 1:**
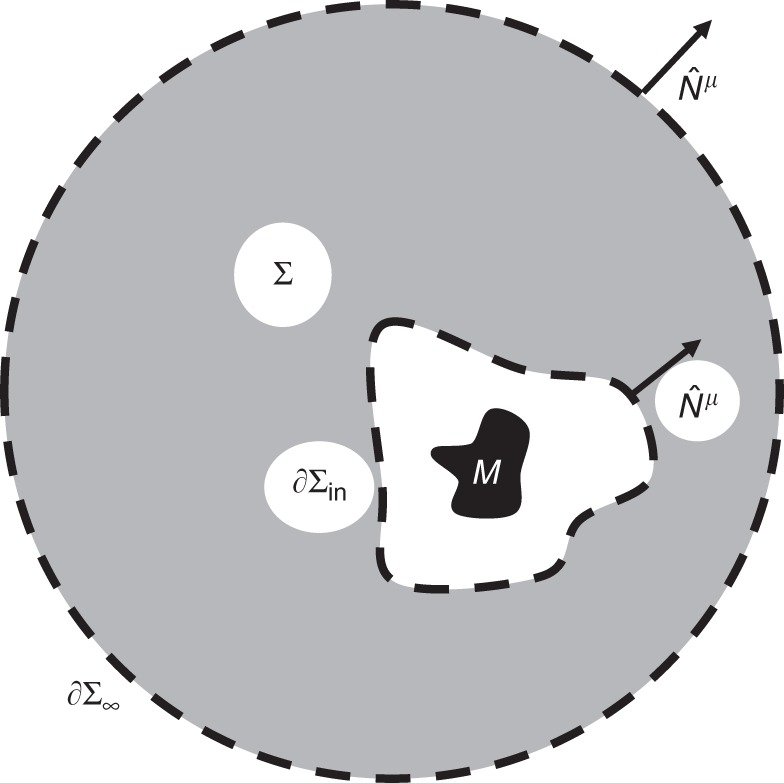
Schematic of the spacelike three-dimensional hypersurface of interest, *Σ*, with an inner boundary ∂*Σ*_in_ and a boundary at infinity *∂Σ*_∞_. Here $\hat N^\mu$ is the spacelike four-vector normal to the boundaries of *Σ* (note the direction convention on the inner boundary). We assume a general mass distribution within the inner boundary and no matter outside it

For a static asymptotically flat spacetime with timelike Killing vector *K*^*μ*^ one may derive the total gravitating mass *M* as an integral over a spacelike hypersurface *Σ* that is truncated (or bounded) internally by an ordinary two-surface ∂*Σ*_in_ (see Fig. 1)

1 $$M = \frac{1}{{4\pi }}{\int}_{ {\hskip -4pt\Sigma }} {\kern 1pt} R_{\mu \nu }K^\mu {\kern 1pt} \hat T^\nu \sqrt {\left| {\gamma ^{({\Sigma })}} \right|} \mathrm{d}^3x + \frac{1}{{4\pi }}{\int}_{\hskip -4pt \partial\Sigma {\varSigma }_{{\mathrm{in}}}} {\kern 1pt} \kappa {\kern 1pt} \mathrm{d}A.$$

(See the Supplementary methods for a detailed derivation and definition of each term.) This expression is a straightforward extension of that used in 1973 by Bardeen et al.^1^ in their derivation of the first law of thermodynamics for black holes, though there the internal boundary was a horizon. Here, *κ* is a natural extension of the surface gravity for nonrotating spacetimes.

### Local temperature

Following the results for horizons^1^, it is tempting to seek to interpret *κ*/(2*π*) from Eq. (1) as the local temperature at any point along an arbitrary two-surface ∂Σ_in_. However, this would be unsatisfactory if true for arbitrary surfaces, since this local temperature would not be in thermal equilibrium with an actual physical screen held fixed at the same location; the temperature now coming from the Unruh effect^3^, and the local proper acceleration required to keep each portion of the screen stationary. Only for surfaces of constant Newtonian gravitational potential *ϕ*, where the proper acceleration of a stationary observer and the local normal to the surface are parallel, is such thermal equilibrium possible (see Supplementary methods). Thus the temptation of such a thermodynamic interpretation should be restricted to the family of ordinary surfaces satisfying *ϕ* = constant.

Indeed, this restricted temptation appears to have been satisfied in the emergent gravity program, where for static asymptotically flat spacetimes, ordinary surfaces of constant *ϕ* are dubbed holographic screens and are claimed to have a local temperature^8^ given by *T* = *κ*/(2*π*), and even to possess a “state variable” quantifying the number of “bits” on the screen. These putative thermodynamic properties are then used in a heuristic derivation of the full Einstein field equations^8^. If correct, such a claim would mean that the emergent gravity program would already subsume many decades of results associated with full general relativity in this setting.

### First law of thermodynamics

Here, we test this thermodynamic assumption by asking whether perturbations of Eq. (1) reproduce the first law of thermodynamics. After all, thermodynamics is primarily a theory about how energy transforms under change, and this aspect of the theory is embodied in the first law. In the simplest case, where the hypersurface *Σ* is empty of matter, this law should read

2 $$\delta M = \frac{1}{{8\pi }}{\int}_{\hskip -4pt \partial {\Sigma }_{{\mathrm{in}}}} {\kern 1pt} \kappa {\kern 1pt} \delta (\mathrm{d}A).$$

We start by following Bardeen et al.’s original analysis^1^, generalizing it where necessary to deal with a boundary *∂Σ*_in_ which is an arbitrary ordinary surface instead of a horizon. Under diffeomorphic metric perturbations we find (see Supplementary methods)

3 $${\delta}{M}=	 \hskip 2pt{\displaystyle\frac{{ - 1}}{{32\pi} }}\displaystyle\int_{\partial {\Sigma }_{{\mathrm{in} }} } [4{\delta}{\theta^{(l)}}+( k_3 + k_6 )\,{\theta}^{(l)} + ( k_3 - k_6 )\,\sigma_{+}^{(l)} \\ 	 {{ + ( {k_4 + k_5} )\,\sigma _ \times^{(l)}} ]\,{\cal N}\mathrm{d}A +{\displaystyle \frac{1}{{8\pi }}}\displaystyle\int_{\partial {\Sigma }_{{\mathrm{in}}}} {\kern 1pt} \kappa {\kern 1pt} \delta (\mathrm{d}A).} $$

Here, *θ*^(*l*)^ and $\sigma _j^{(l)}$ (*j* = +, ×) are the expansion and shears of null normal congruences of geodesics, the change in the expansion under the diffeomorphism is given by

4 $$\delta \theta ^{(l)} = - \frac{{k_2}}{2}\theta ^{(l)} + \frac{1}{2}\left( {k_3 + k_6} \right)_{;\rho }\hat N^\rho ,$$

and the *k*_*j*_ are functions corresponding to independent components of the metric perturbation. As the expansion and shears vanish identically on the horizon^9^, we see that Eq. (3) trivially reduces to the first law, Eq. (2), thus reproducing the famous 1973 result^1^. Similarly, it follows straightforwardly that for surfaces sufficiently close to the horizon (so-called stretched horizons), the corrections to the first law will be negligible.

### Surfaces away from horizons

So far we have assumed that the inner boundaries before and after the diffeomorphic perturbation are arbitrary. But could the perturbed boundary be chosen in a specific manner so as to cause the unwanted terms in Eq. (3) to vanish? As already noted, holographic screens correspond to surfaces of constant Newtonian potential *ϕ* = constant. Thus, the perturbed screen relies on a specification of the constant *δϕ*. It is easy to show that $\delta \phi = \frac{1}{2}k_1$ (see Supplementary methods), where *k*_1_ is a metric perturbation of which the unwanted terms in Eq. (3) are wholly independent. Thus, the ordinary surfaces used within the emergent gravity program cannot generally satisfy the first law, Eq. (2).

One caveat to this claim comes when we consider a fully spherically symmetric scenario; where both the initial spacetime and screen are spherically symmetric, so the initial shears $\sigma _j^{(l)}$ vanish, and also the final spacetime and screen are spherically symmetric, placing further constraints on the *k*_*j*_. In this case, Birkhoff’s theorem^10^ for spherically symmetric metrics imposes extra constraints between the metric components so that a perturbed screen may always be chosen so as to satisfy the form of the first law^11^. However, as noted above, this form will not be preserved under arbitrary metric perturbations.

## Discussion

The implications of our results are now described for (i) stretched horizons, and (ii) ordinary surfaces

(i) Stretched horizons have long been considered to act as black bodies^6^, effectively radiating with a temperature *κ*/(2*π*). Thus, our demonstration that they also satisfy the first law to an excellent approximation hardly seems surprising. Nevertheless, we do not believe that our result here should be interpreted as implying that the surfaces corresponding to stretched horizons themselves should be imbued with actual thermodynamic properties.

In particular, we may consider an alternative spacetime, identical from the stretched horizon outward, but instead of a horizon, we consider an infinitesimal shell of matter just outside what would correspond to its Schwarzschild radius were the shell to collapse further, yet still within the “stretched horizon”. In this latter spacetime, there is no horizon and hence no Hawking radiation. Notwithstanding this, our work proves that the “stretched horizon” still closely satisfies the first law.

We conclude from this that the laws of black-hole mechanics are not sufficient in themselves to guarantee whether any particular surface is truly thermodynamic in nature. For stretched horizons, we interpret this reasoning to imply that their full thermodynamic behavior is only inherited from the presence of an underlying horizon, but is not intrinsic to stretched horizons themselves. This conclusion appears to mimic the initial reluctance of general relativists^1^ from accepting black-hole horizons as truly thermodynamic despite the deep analogy to thermodynamics uncovered in the laws of black-hole mechanics. By contrast, these laws should still be considered a necessary condition.

(ii) Our analysis further rigorously shows that the family of ordinary surfaces called holographic screens will generally not obey a first law of thermodynamics, in contrast to the long-standing result for horizons^1^. (Other families would not even be in thermal equilibrium with a physical surface at the same location.) Recall that the first law is more general than thermodynamics: the “temperature” is merely an integrating factor relating changes in energy to changes in some state variable (entropy in the case of thermodynamics). Failure of the first law means that the putative state variable is not a variable of state at all. Therefore, even in static asymptotically flat spacetimes, where the emergent gravity program claims to derive the full Einstein field equations, our results show that the key assumption of this program is actually inconsistent with general relativity.

## Methods

### Energy conservation under diffeomorphisms

In order to attempt to derive a first law for ordinary surfaces we closely follow in the footsteps of Bardeen et al.’s 1973 classic paper^1^. The first step is to obtain an integral equation for the net energy in a static system, Eq. (1), where instead of an inner boundary located at a black-hole horizon, this boundary is an ordinary surface. Next, we consider small “changes” in the net energy corresponding to shifting to a parametrically nearly solution to the Einstein field equations. This “differential” version is determined by studying the behavior of the net energy under spacetime diffeomorphisms of the initial metric^1^. As in Bardeen et al., “gauge” freedom in the choice of coordinates is used to ensure that the hypersurfaces before and after the diffeomorphism are covered by identical sets of coordinates.

### Study assumptions

Our analysis is limited to static asymptotically flat solutions, with zero shift vector, *β*^*μ*^ = 0. For simplicity, we assume that the spacetime of interest is nonrotating, and that there is no matter exterior to the holographic screen (*T*^*μν*^ = 0). We work throughout in natural units where *G* = *c* = *ħ* = *k*_*B*_ = 1. Full and extensive details of the analysis are provided in the Supplementary methods.

### Data availability

The authors declare that all relevant data of this study are contained in the article and its Supplementary information

## Electronic supplementary material

Supplementary Information

## References

[1] Bardeen JM, Carter B, Hawking SW (1973). The four laws of black-hole mechanics. Commun. Math. Phys..

[2] Hawking SW (1975). Particle creation by black holes. Commun. Math. Phys..

[3] Unruh WG (1976). Notes on black-hole evaporation. Phys. Rev. D.

[4] Jacobson T (1995). Thermodynamics of spacetime: the Einstein equation of state. Phys. Rev. Lett..

[5] Gibbons GW, Hawking SW (1977). Cosmological event horizons, thermodynamics, and particle creation. Phys. Rev. D.

[6] Thorne KS, Price RH, Macdonald DA (1986). Black Holes: The Membrane Paradigm.

[7] Zurek WH, Thorne KS (1985). Statistical mechanical origin of the entropy of a rotating, charged black-hole. Phys. Rev. Lett..

[8] Verlinde E (2011). On the Origin of Gravity and the Laws of Newton. J. High Energy Phys..

[9] Hawking, S. W. & Ellis, G. F. R. *The Large Scale Structure of Spacetime* 82–83, p. 334 (Cambridge University Press, 1973).

[10] Birkhoff GD, Langer RE (1923). Relativity and Modern Physics.

[11] Chen YX, Li JL (2011). First law of thermodynamics on holographic screens in entropic force frame. Phys. Lett. B.

