# Supplementary material for: Surfaces away from horizons are not thermodynamic
Source: Nat Commun. 2018 Jul 30;9:2977. doi: 10.1038/s41467-018-05433-9 (PMC6065406; doi:10.1038/s41467-018-05433-9)
Supplement: Supplementary file 1 — Supplementary Information [file 41467_2018_5433_MOESM1_ESM.pdf]

Supplementary Information

for

“Surfaces away from horizons are not thermodynamic”

Wang and Braunstein

Supplementary information

Zhi-Wei Wang[1,2] and Samuel L. Braunstein[2]
[1] *College of Physics, Jilin University, Changchun, 130012, People's Republic of China and*
[2] *Computer Science, University of York, York YO10 5GH, United Kingdom* *
(Dated: June 27, 2018)

SUPPLEMENTARY METHODS

Introduction

In order to attempt to derive a first law for ordinary surfaces within a spacetime we shall follow closely in the footsteps of Bardeen, Carter and Hawking's 1973 classic paper [1]. The first step is to obtain an integral equation for the net energy in a static system, where instead of an inner boundary located at a black hole horizon, this boundary will be an ordinary surface. In order to obtain a first law, we must convert this into a description of how small "changes" in the net energy may be accounted for via a differential version of energy balance. The changes here actually refer to the differences between nearby solutions of the field equations related by a small diffeomorphism of the initial metric. Of course such changes could be implemented physically via an adiabatic process involving weightless strings used to add, subtract, or otherwise rearrange matter within the screen. Alternatively, one might consider physical changes due to matter dynamically falling past the screen [2], but we shall *not* study this latter approach here. We set $G = c = \hbar = k_B = 1$ throughout.

As already noted, we shall be considering diffeomorphisms between pairs of nearby static asymptotically-flat solutions with zero shift vector ($\beta^\mu = 0$). For simplicity, we shall suppose (from Eq. (10) onwards) that the spacetime is non-rotating. Finally, from Eq. (20) onwards, we shall assume that there is no matter *exterior* to the inner boundary ($T^{\mu\nu} = 0$). For ease of navigation, we have placed in boxes all the key results used in the main manuscript.

The original 1973 paper [1] gives key signposts for deriving the first law. However, we have been unable to find a *detailed* derivation in any subsequent paper or textbook. Therefore, other than the above simplifications (which suffice for our purposes in studying ordinary surfaces) we give those details here. This is done partly to allow our claims to be checked in detail and partly as a resource for the community to better appreciate the original 1973 result.

We focus specifically on generalizing the first law of black hole mechanics to ordinary surfaces. Indeed, it is straightforward to derive an integral version of the first law associated with arbitrary surfaces. This allows one to identify how surface gravity (and hence presumably temperature) should be generalized away from a horizon in a manner consistent with the natural generalization of the area law. Next, following Bardeen et al., we study diffeomorphisms of this integral formulation in order to attempt to construct a differential first law. Everything follows through as in the 1973 analysis except that diffeomorphisms of the surface gravity of an ordinary surface does not reduce to the form found for horizons. We conclude that the first law of thermodynamics does not hold for ordinary 2-surfaces $\partial\Sigma_{\rm in}$ and hence such surfaces do not behave thermodynamically.

We finish this introduction with a list of key symbols:

M	mass
$\mathcal{N}$	lapse function ($= 1/\hat{T}^t$)
β^μ	shift vector (= 0 throughout)
$g_{\mu\nu}$	metric
$T^{\mu\nu}$	energy-momentum tensor (= 0 from Eq.(20))
$R_{\mu\nu}$	curvature tensor
R	Ricci scalar
Σ	3-dimensional hypersurface in this paper
$\Sigma_{\mathbf{EG}}$	3-dimensional hypersurface in Ref. 4
$\partial\Sigma$	boundary of Σ
$\partial\Sigma_\infty$	outer boundary of Σ
$\partial\Sigma_{\mathbf{in}}$	inner boundary of Σ (ordinary surface)
$\partial\Sigma_{\mathbf{BH}}$	inner boundary of Σ (black hole horizon)
$\partial\Sigma_{\mathbf{HS}}$	inner boundary of Σ (holographic screen)
$\gamma^{(\Sigma)}$	induced metric on Σ
$\gamma^{(\partial\Sigma)}$	induced metric on $\partial\Sigma$
dA	area element ($= \sqrt{\gamma^{(\partial\Sigma)}}\, dy^2$)
$\hat{T}^\mu$	timelike unit normal vector of Σ
$\hat{N}^\mu$	spacelike unit normal vector of $\partial\Sigma$
κ	generalized surface gravity, Eq. (7)
K^μ	Killing vector of the static spacetime ($= \partial_t$)
$h_{\mu\nu}$	diffeomorphic variation of the metric ($= \delta g_{\mu\nu}$)
$\theta^{(l)}$	expansion of the outgoing null normal vector l^μ
$\sigma_+^{(l)}$, $\sigma_\times^{(l)}$	shears of the outgoing null normal vector l^μ
$\sigma_P^{(l)}$	'principle shear' of the outgoing null normal vector l^μ

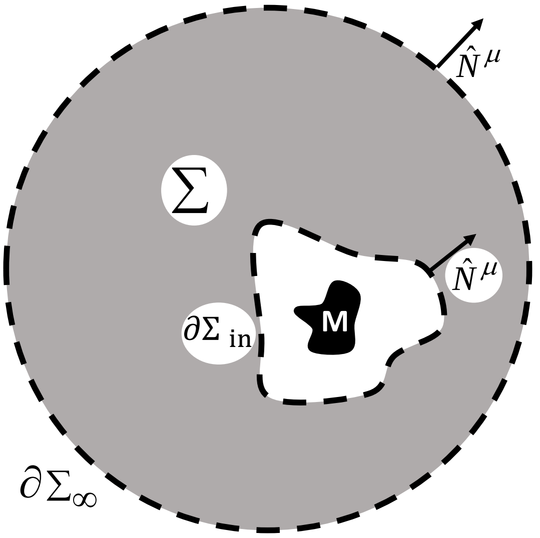

FIG. 1: Schematic of the spacelike three-dimensional hypersurface of interest, Σ, with an inner boundary $\partial\Sigma_{\text{in}}$ and a boundary at infinity $\partial\Sigma_\infty$. Here $\hat{N}^\mu$ is the spacelike 4-vector normal to the boundaries of Σ (note the direction convention on the inner boundary). In the absence of matter outside the inner boundary, the gravitating mass as measured at infinity, M, will be entirely inside the inner boundary.

Integral expression for net energy

Consider a static spacetime with a Killing vector $K^\mu = \partial_t = (1,0,0,0)$, with $K^\mu K_\mu = -1$ at spatial infinity. The Killing equation implies that

$$K_{\mu;\nu} = K_{[\mu;\nu]} \equiv \frac{1}{2}(K_{\mu;\nu} - K_{\nu;\mu}) \ . \quad (1)$$

Now recall that permuting the order of a pair of covariant derivatives acting on a 4-vector A^μ may be expressed in terms of the Riemann curvature tensor as [3]

$$A^\mu{}_{;\alpha\beta} - A^\mu{}_{;\beta\alpha} = -R^\mu{}_{\nu\alpha\beta}A^\nu \ .$$

Contracting the indices μ and α reduces this to an expression in terms of the Ricci tensor

$$A^\mu{}_{;\mu\beta} - A^\mu{}_{;\beta\mu} = -R_{\nu\beta}A^\nu \ . \quad (2)$$

Since the Killing vector is anti-symmetric we must have $K^\mu{}_{;\mu} = 0$ and we immediately find that

$$K^\mu{}_{;\beta\mu} = R_{\nu\beta}K^\nu. \quad (3)$$

Integrating this over a spacelike hypersurface Σ, yields

$$\int_\Sigma K^\mu{}_{;\beta\mu}\,\hat{T}^\beta\,\sqrt{|\gamma^{(\Sigma)}|}\,d^3x = \int_\Sigma R_{\nu\beta}K^\nu\,\hat{T}^\beta\,\sqrt{|\gamma^{(\Sigma)}|}\,d^3x \quad (4)$$

here $\hat{T}^\mu$ is the timelike unit 4-vector normal to Σ, so $\hat{T}^\mu\hat{T}_\mu = -1$.

The hypersurface is assumed to have an outer boundary at spatial infinity $\partial\Sigma_\infty$, and an inner boundary Σ_{in} (see Fig. 1). In the original work of Bardeen et al. [1], this inner boundary corresponded to the black hole's horizon $\partial\Sigma_{\text{BH}}$. Here we generalize this by taking it to be an arbitrary closed 2-surface $\partial\Sigma_{\text{in}}$. The boundary of the hypersurface is assumed to be oriented, with unit normal $\hat{N}^\mu$ (see Fig. 1), so $\hat{N}_\mu\hat{N}^\mu = 1$ and $\hat{N}^\mu\hat{T}_\mu = 0$.

Recalling Stokes's theorem for an anti-symmetric tensor $F^{\mu\nu}$ [3]

$$\int_\Sigma \hat{T}_\mu F^{\mu\nu}{}_{;\nu}\sqrt{|\gamma^{(\Sigma)}|}dx^{n-1} = \int_{\partial\Sigma} \hat{T}_\mu F^{\mu\nu}\hat{N}_\nu\sqrt{|\gamma^{(\partial\Sigma)}|}dy^{n-2}, \quad (5)$$

and applying it to the left-hand-side of Eq. (4) we find

$$\int_{\partial\Sigma_\infty} K^\mu{}_{;\beta}\,\hat{T}^\beta\hat{N}_\mu\sqrt{|\gamma^{(\partial\Sigma_\infty)}|}\,d^2y - \int_{\partial\Sigma_{\text{in}}} K^\mu{}_{;\beta}\,\hat{T}^\beta\,\hat{N}_\mu\,\sqrt{|\gamma^{(\partial\Sigma_{\text{HS}})}|}\,d^2y = \int_\Sigma R_{\nu\beta}K^\nu\,\hat{T}^\beta\,\sqrt{|\gamma^{(\Sigma)}|}\,d^3x. \quad (6)$$

At this stage, we wish to generalize the concept of surface gravity as a quantity defined anywhere. Assuming that the surface $\partial\Sigma$ is non-rotating (corresponding to zero angular velocity of the spacetime itself) , we may interpret the integrand of the integral on the boundary in Eq. (6) to be the surface gravity, so

$$\boxed{\kappa \equiv K^\mu{}_{;\nu}\hat{T}^\nu\hat{N}_\mu.} \quad (7)$$

It is worth noting that $\kappa/(2\pi)$ is precisely the formula Verlinde gives (his Eq. (5.3) of [4]) for what he calls the local temperature of the holographic screen (ordinary surfaces of constant Newtonian potential ϕ) as measured with respect to a reference point at spatial infinity.

This definition of surface gravity allows us to naturally extend the original 1973 analysis away from black hole horizons. In particular, the left-hand-side of Eq. (6) reduces to

$$\int_{\partial\Sigma_\infty} \kappa\,\sqrt{|\gamma^{(\partial\Sigma_\infty)}|}\,d^2y - \int_{\partial\Sigma_{\text{in}}} \kappa\,\sqrt{|\gamma^{(\partial\Sigma_{\text{HS}})}|}\,d^2y. \quad (8)$$

The integral over $\partial\Sigma_\infty$ reduces to the Komar expression

for the *total* gravitating mass within the system, M, [3] leading to

$$\boxed{M = \frac{1}{4\pi}\int_{\Sigma} R_{\mu\nu} K^{\mu}\,\hat{T}^{\nu}\,\sqrt{|\gamma^{(\Sigma)}|}\,d^3x + \frac{1}{4\pi}\int_{\partial\Sigma_{\rm in}} \kappa\, dA\ ,} \tag{9}$$

Were we to consider spherically symmetric case, Eq. (9) would reduce to

$$M = \frac{1}{4\pi}\int_{\Sigma} R_{\mu\nu} K^{\mu}\,\hat{T}^{\nu}\,\sqrt{|\gamma^{(\Sigma)}|}\,d^3x + \frac{\kappa}{4\pi}A. \tag{10}$$

(This is exactly the first law given by Bardeen et al. [1] taking angular velocity of $\partial\Sigma$ to vanish).

Just to emphasize what this represents, here the hypersurface, Σ, extends from an arbitrary inner boundary, $\partial\Sigma_{\rm in}$, out to spatial infinity. Thus, the generalized surface gravity, κ, and the area, A, are those associated with the inner boundary itself (rather than any horizon).

Eq. (9) has exactly the same form as the conventional formula for the total mass of the system [1] but extended to an arbitrary 2-dimensional surface (instead of a horizon). Finally, note that the matter inside the inner boundary need *not* be associated with a black hole, it may be ordinary matter, with no horizon present at all. Thus, were inner boundaries found to have thermodynamic properties (i.e., a well-defined entropy and temperature), it would *not* be because such properties were inherited from a real horizon behind the screen.

Differential "first law"

The above straightforward generalization, especially in the spherically symmetric case, for net energy on a hypersurface might appear to suggest that a temperature and entropy can actually be defined for any surface by

$$T = \frac{\kappa}{2\pi}, \qquad S = \frac{A}{4}. \tag{11}$$

However, such quantities need to behave thermodynamically. In particular, for our static system, the net energy E, should admit changes which behave as

$$\delta E = T\delta S, \tag{12}$$

(ignoring work terms) so that the temperature would be acting as an integrating factor relating changes in the (state function) entropy to changes in the energy. In other words, we must show that such changes lead to the expected form of the first-law of thermodynamics. Again here we follow in the footsteps of the original analysis and consider changes corresponding to parametric differences between diffeomorphicly nearby solutions. In particular, we will consider two nearby configurations corresponding to the metrics

$$g_{\mu\nu}, \qquad g'_{\mu\nu} = g_{\mu\nu} + h_{\mu\nu}, \tag{13}$$

where $h_{\mu\nu} \equiv \delta g_{\mu\nu} = -g_{\mu\sigma}g_{\nu\tau}\delta g^{\sigma\tau}$, i.e., $\delta g^{\sigma\tau} = -h^{\sigma\tau}$.

As with the original analysis and without loss of generality, we may assume that for the two diffeomorphicly related configurations, the hypersurfaces Σ and Σ' are described by identical sets of coordinates; this is always possible due to "gauge" freedom in the choice of coordinate systems [1]. Henceforth we label both by Σ. Similarly, for their boundaries $\partial\Sigma$. Further, as in [1] we likewise assume that both configurations have the same Killing vector, so

$$\delta K^{\mu} = 0, \qquad \delta K_{\mu} = h_{\mu\nu}K^{\nu}. \tag{14}$$

Finally, it will be sufficient for our purposes to consider only the case where there is no matter on Σ itself, so $T^{\mu\nu} = 0$ there. Geometrically, this corresponds to all the matter lying behind or within the inner boundary $\partial\Sigma_{\rm in}$ (see Fig. 1).

In order to consider diffeomorphisms which need not respect spherical symmetry, we return to Eq. (9). Using the Einstein field equations we start by rewriting this integral formula as

$$M = \int_{\Sigma}(2\,T_{\mu\nu} + \frac{1}{8\pi}R\,g_{\mu\nu})K^{\mu}\,\hat{T}^{\nu}\,\sqrt{|\gamma^{(\Sigma)}|}\,d^3x + \frac{1}{4\pi}\int_{\partial\Sigma_{\rm in}} \kappa\, dA\ , \tag{15}$$

Recall $K^{\mu} = \partial_t$ and $\hat{T}^{\mu}$ is normal to Σ, so $K^{\mu} = \mathcal{N}\hat{T}^{\mu} + \beta^{\mu}$ where β^{μ} is the shift vector and $\mathcal{N} = 1/\hat{T}^t$ is the lapse function [5]. Assuming a zero shift vector $\beta^{\mu} = 0$, then $\hat{T}^{\mu} = \hat{T}^t K^{\mu}$ and $\hat{T}_{\mu} = \hat{T}^t K_{\mu}$. Since $\mathcal{N}\sqrt{|\gamma^{(\Sigma)}|} = \sqrt{-g}$ on the

hypersurface [5], the variation of the Ricci scalar term may be computed as

$$\begin{aligned}
& \frac{1}{8\pi}\int_\Sigma \delta(R\sqrt{|\gamma^{(\Sigma)}|}\,K^\beta\,\hat{T}_\beta)\,d^3x \\
=\ & \frac{1}{8\pi}\int_\Sigma \delta(R\mathcal{N}\sqrt{|\gamma^{(\Sigma)}|}\,\hat{T}^\beta\,\hat{T}_\beta)\,d^3x \\
=\ & \frac{1}{8\pi}\int_\Sigma \delta(R\sqrt{-g})\,\hat{T}^\beta\,\hat{T}_\beta\,d^3x \\
=\ & -\frac{1}{8\pi}\int_\Sigma \Big((R_{\mu\nu}-\frac{1}{2}g_{\mu\nu}R)h^{\mu\nu} - (g^{\mu\nu}\delta\Gamma^\alpha{}_{\mu\nu} - g^{\mu\alpha}\delta\Gamma^\lambda{}_{\lambda\mu})_{;\alpha}\Big)\,K^\beta\,\hat{T}_\beta\,\sqrt{|\gamma^{(\Sigma)}|}\,d^3x\,. \qquad (16)
\end{aligned}$$

where in the last step we have used the well-known result that [6]

$$\delta(R\sqrt{-g}) = -\Big((R_{\mu\nu}-\frac{1}{2}g_{\mu\nu}R)h^{\mu\nu} - (g^{\mu\nu}\delta\Gamma^\alpha{}_{\mu\nu} - g^{\mu\alpha}\delta\Gamma^\lambda{}_{\lambda\mu})_{;\alpha}\Big)\sqrt{-g}. \qquad (17)$$

Lemma 1: $-(g^{\mu\nu}\delta\Gamma^\alpha{}_{\mu\nu} - g^{\mu\alpha}\delta\Gamma^\lambda{}_{\lambda\mu})_{;\alpha} = 2\,h^\mu{}_{[\mu;\nu]}{}^{;\nu}$, a result quoted in Ref. [1], there without proof.
Proof:
Since [7]

$$\delta\Gamma^\alpha_{\mu\nu} = \frac{1}{2}g^{\alpha\rho}(h_{\mu\rho;\nu} + h_{\nu\rho;\mu} - h_{\mu\nu;\rho})\,, \qquad (18)$$

we have

$$\begin{aligned}
-(g^{\mu\nu}\delta\Gamma^\alpha{}_{\mu\nu} - g^{\mu\alpha}\delta\Gamma^\lambda{}_{\lambda\mu})_{;\alpha} &= \Big(g^{\mu\alpha}\frac{1}{2}g^{\lambda\rho}(h_{\mu\rho;\lambda} + h_{\lambda\rho;\mu} - h_{\mu\lambda;\rho}) - g^{\mu\nu}\frac{1}{2}g^{\alpha\rho}(h_{\mu\rho;\nu} + h_{\nu\rho;\mu} - h_{\mu\nu;\rho})\Big)_{;\alpha} \\
&= \frac{1}{2}\Big(g^{\mu\alpha}h^\rho{}_{\rho;\mu} - g^{\mu\nu}(h^\alpha{}_{\mu;\nu} + h^\alpha{}_{\nu;\mu} - h_{\mu\nu}{}^{;\alpha})\Big)_{;\alpha} \\
&= \frac{1}{2}\Big(h_\rho{}^{\rho;\alpha} - (h^{\alpha\nu}{}_{;\nu} + h^{\alpha\mu}{}_{;\mu} - h_\mu{}^{\mu;\alpha})\Big)_{;\alpha} \\
&= \frac{1}{2}(2\,h_\rho{}^{\rho;\alpha} - 2\,h^{\alpha\mu}{}_{;\mu})_{;\alpha} \\
&= 2\,h^\mu{}_{[\mu;\nu]}{}^{;\nu}. \qquad (19)
\end{aligned}$$

This completes the proof of Lemma 1. □

Using Lemma 1, the variation of the Ricci scalar term becomes

$$-\frac{1}{8\pi}\int_\Sigma\Big((R_{\mu\nu}-\frac{1}{2}g_{\mu\nu}R)h^{\mu\nu} + 2h^\mu{}_{[\mu;\nu]}{}^{;\nu}\Big)\,K^\beta\,\hat{T}_\beta\,\sqrt{|\gamma^{(\Sigma)}|}\,d^3x = -\frac{1}{4\pi}\int_\Sigma h^\mu{}_{[\mu;\nu]}{}^{;\nu}K^\beta\,\hat{T}_\beta\,\sqrt{|\gamma^{(\Sigma)}|}\,d^3x \qquad (20)$$

since the first term is zero if we assume $T^{\mu\nu} = 0$ on Σ outside the holographic screen.

Lemma 2: $h^\mu{}_{[\mu;\nu]}{}^{;\nu}K^\beta = (K^\beta h_\mu{}^{[\mu;\nu]} - K^\nu h_\mu{}^{[\mu;\beta]})_{;\nu}$, a result quoted in Ref. [1], there without proof.

Proof: Expanding out the right-hand-side (rhs) of the claim in Lemma 2, we get

$$\text{rhs} = h_\mu{}^{[\mu;\nu]}{}_{;\nu}K^\beta + h_\mu{}^{[\mu;\nu]}K^\beta{}_{;\nu} - h_\mu{}^{[\mu;\beta]}{}_{;\nu}K^\nu\,, \qquad (21)$$

since $h_\mu{}^{[\mu;\nu]}{}_{;\nu}K^\beta = h^\mu{}_{[\mu;\nu]}{}^{;\nu}K^\beta$, Eq. (21) reduces to

$$h^\mu{}_{[\mu;\nu]}{}^{;\nu}K^\beta + h_\mu{}^{[\mu;\nu]}K^\beta{}_{;\nu} - h_\mu{}^{[\mu;\beta]}{}_{;\nu}K^\nu = h^\mu{}_{[\mu;\nu]}{}^{;\nu}K^\beta - \pounds_K(h_\mu{}^{[\mu;\beta]}) = h^\mu{}_{[\mu;\nu]}{}^{;\nu}K^\beta = \text{lhs}\,. \qquad (22)$$

The Lie derivative along K^μ vanishes since the pair of diffeomorphicly related metrics are assumed static [8]. This completes the proof of Lemma 2. □

Applying Lemma 2 to Eq. (20), the variation of the term involving the Ricci scalar reduces to

$$-\frac{1}{4\pi}\int_{\Sigma}(K^{\beta}h_{\mu}{}^{[\mu;\nu]}-K^{\nu}h_{\mu}{}^{[\mu;\beta]})_{;\nu}\,\hat{T}_{\beta}\,\sqrt{|\gamma^{(\Sigma)}|}\,d^3x. \tag{23}$$

Thus, the variation in the total mass may be written

$$\delta M=-\frac{1}{4\pi}\int_{\Sigma}(K^{\beta}h_{\mu}{}^{[\mu;\nu]}-K^{\nu}h_{\mu}{}^{[\mu;\beta]})_{;\nu}\,\hat{T}_{\beta}\,\sqrt{|\gamma^{(\Sigma)}|}\,d^3x+\frac{1}{4\pi}\int_{\partial\Sigma_{\rm in}}\delta\kappa\,dA+\frac{1}{4\pi}\int_{\partial\Sigma_{\rm in}}\kappa\,\delta(dA). \tag{24}$$

Since the term inside the bracket is an anti-symmetric tensor, we may use Stokes's theorem, Eq. (5), to obtain

$$\begin{aligned}\delta M=\;&-\frac{1}{4\pi}\int_{\partial\Sigma_{\infty}}(K^{\beta}h_{\mu}{}^{[\mu;\nu]}-K^{\nu}h_{\mu}{}^{[\mu;\beta]})\hat{N}_{\nu}\,\hat{T}_{\beta}\,\sqrt{|\gamma^{(\partial\Sigma_{\infty})}|}\,d^2y\\&+\frac{1}{4\pi}\int_{\partial\Sigma_{\rm in}}(K^{\beta}h_{\mu}{}^{[\mu;\nu]}-K^{\nu}h_{\mu}{}^{[\mu;\beta]})\hat{N}_{\nu}\,\hat{T}_{\beta}\,\sqrt{|\gamma^{(\partial\Sigma_{\rm in})}|}\,d^2y+\frac{1}{4\pi}\int_{\partial\Sigma_{\rm in}}\delta\kappa\,dA+\frac{1}{4\pi}\int_{\partial\Sigma_{\rm in}}\kappa\,\delta(dA),\end{aligned} \tag{25}$$

where the boundary has be split into the inner boundary $\partial\Sigma_{\rm in}$ and the boundary at infinity $\partial\Sigma_{\infty}$. The contribution for the term at infinity may be evaluated using the notation of tensorial volume elements [9] as

$$\begin{aligned}-\frac{1}{4\pi}\int_{\partial\Sigma_{\infty}}(K^{\beta}h_{\mu}{}^{[\mu;\nu]}-K^{\nu}h_{\mu}{}^{[\mu;\beta]})\hat{N}_{\nu}\,\hat{T}_{\beta}\,\sqrt{|\gamma^{(\partial\Sigma_{\infty})}|}\,d^2y\;&=\;-\frac{1}{4\pi}\int_{\partial\Sigma_{\infty}}K^{\beta}h_{\mu}{}^{[\mu;\nu]}(\hat{T}_{\beta}\,\hat{N}_{\nu}\,-\hat{T}_{\nu}\,\hat{N}_{\beta})\sqrt{|\gamma^{(\partial\Sigma_{\infty})}|}\,d^2y\\&=\;-\frac{1}{4\pi}\int_{\partial\Sigma_{\infty}}K^{\beta}h_{\mu}{}^{[\mu;\nu]}\,\varepsilon_{\beta\nu}\,\varepsilon_{\alpha\mu}\\&=\;\frac{1}{8\pi}\int_{\partial\Sigma_{\infty}}(h_{\mu}{}^{\mu;\nu}-h_{\mu}{}^{\nu;\mu})K^{\beta}\,\varepsilon_{\beta\nu\alpha\mu}\\&=\;-\delta M\;,\end{aligned} \tag{26}$$

where the orientation of $\varepsilon_{\beta\nu\alpha\mu}$ is chosen so that $\varepsilon_{\beta\nu\alpha\mu}=-6\,\varepsilon_{[\beta\nu}\varepsilon_{\alpha\mu]}$ and $\varepsilon_{\alpha\mu}$ is the volume element of the boundary at infinity, and we have applied the result $\frac{1}{8\pi}\int_{\partial\Sigma_{\infty}}(h_{\mu}{}^{\mu;\nu}-h_{\mu}{}^{\nu;\mu})K^{\beta}\,\varepsilon_{\beta\nu\alpha\mu}=-\delta M$ in the final step [9].

Eq. (26) allows us to transform Eq. (25) into

$$\delta M=-\delta M+\frac{1}{4\pi}\int_{\partial\Sigma_{\rm in}}(K^{\beta}h_{\mu}{}^{[\mu;\nu]}-K^{\nu}h_{\mu}{}^{[\mu;\beta]})\hat{N}_{\nu}\,\hat{T}_{\beta}\,\sqrt{|\gamma^{(\partial\Sigma_{\rm in})}|}\,d^2y+\frac{1}{4\pi}\int_{\partial\Sigma_{\rm in}}\delta\kappa\,dA+\frac{1}{4\pi}\int_{\partial\Sigma_{\rm in}}\kappa\,\delta(dA). \tag{27}$$

Or equivalently,

$$\delta M=\frac{1}{8\pi}\int_{\partial\Sigma_{\rm in}}\frac{1}{2}(h_{\mu}{}^{\mu;\nu}-h_{\mu}{}^{\nu;\mu})\hat{N}_{\nu}\,\hat{T}_{\beta}K^{\beta}\,dA+\frac{1}{8\pi}\int_{\partial\Sigma_{\rm in}}\delta\kappa\,dA+\frac{1}{8\pi}\int_{\partial\Sigma_{\rm in}}\kappa\,\delta(dA)\;, \tag{28}$$

where we have used $K^{\nu}\hat{N}_{\nu}=\mathcal{N}\hat{T}^{\nu}\hat{N}_{\nu}=0$, which follows since $\hat{T}^{\mu}$ is normal to Σ and $\hat{N}^{\mu}$ lies in Σ.

For the first law to be true, we would require that the first two boundary integrals of Eq. (28) exactly cancel. In order to further simplify these terms we start by considering the diffeomorphic changes in more detail.

Diffeomorphic conditions

As already discussed, we assume

$$\delta K^{\mu}=0,\qquad\qquad \delta K_{\mu}=h_{\mu\nu}K^{\nu}. \tag{29}$$

Recall that by "gauge" freedom the sets of coordinates of Σ and $\partial\Sigma$ are unchanged by the diffeomorphism, so without loss of generality we may take [1]

$$\delta(dx^{\mu})=0\;,\forall dx^{\mu}\text{ in }\Sigma. \tag{30}$$

Because $K_{\mu}dx^{\mu}=\mathcal{N}\hat{T}_{\mu}dx^{\mu}=0$ for all dx^{μ} in Σ, we have $\delta K_{\mu}\parallel K_{\mu}$, so

$$\delta K_{\mu}=k_0K_{\mu}. \tag{31}$$

Comparing Eq. (31) with Eq. (29), one finds

$$h_{\mu\nu}K^{\nu}=k_0K_{\mu} \tag{32}$$

everywhere. Then contracting $\hat{T}^{\mu}$ on both sides of this equation yields

$$k_0=-h_{\mu\nu}\hat{T}^{\mu}\hat{T}^{\nu}. \tag{33}$$

(In other words, $k_0 = -h_{\hat{T}\hat{T}}$ in the tetrad basis.)

Similarly, since $\hat{T}_\mu dx^\mu = 0$ for all dx^μ in Σ, we have $\delta\hat{T}_\mu \parallel \hat{T}_\mu$, so

$$\delta\hat{T}_\mu = \frac{k_1}{2}\hat{T}_\mu \tag{34}$$

(the factor of $\frac{1}{2}$ is for later convenience). To get an expression for k_1, we calculate the variation of $g^{\mu\nu}\hat{T}_\mu\hat{T}_\nu = -1$.

$$\begin{aligned} (\delta g^{\mu\nu})\hat{T}_\mu\hat{T}_\nu + 2g^{\mu\nu}\hat{T}_\mu(\delta\hat{T}_\nu) &= 0 \\ \Rightarrow \quad (-h^{\mu\nu})\hat{T}_\mu\hat{T}_\nu + 2g^{\mu\nu}\hat{T}_\mu(\frac{k_1}{2}\hat{T}_\nu) &= 0, \end{aligned} \tag{35}$$

hence

$$k_1 = -h_{\mu\nu}\hat{T}^\mu\hat{T}^\nu = k_0. \tag{36}$$

For $\hat{T}^\mu$, we find

$$\begin{aligned} \delta\hat{T}^\mu &= \delta(g^{\mu\nu}\hat{T}_\nu) \\ &= -h^{\mu\nu}\hat{T}_\nu + \frac{1}{2}k_1\hat{T}^\mu \\ &= -g^{\mu\lambda}h_{\lambda\nu}\hat{T}^\nu + \frac{1}{2}k_1\hat{T}^\mu \\ &= -g^{\mu\lambda}h_{\lambda\nu}K^\nu\hat{T}^t + \frac{1}{2}k_1\hat{T}^\mu \\ &= -g^{\mu\lambda}k_1K_\lambda\hat{T}^t + \frac{1}{2}k_1T^\mu \\ &= -k_1\hat{T}^\mu + \frac{1}{2}k_1\hat{T}^\mu \\ &= -\frac{1}{2}k_1\hat{T}^\mu, \end{aligned} \tag{37}$$

where we have used Eq. (32) in the fourth line.

Again since, $dx^\mu\hat{N}_\mu = 0$ for all dx^μ in $\partial\Sigma$, combined with $\hat{T}^\mu\hat{N}_\mu = 0$, we find $\delta\hat{N}_\mu \parallel \hat{N}_\mu$, and so we write

$$\delta\hat{N}_\mu = \frac{1}{2}k_2\hat{N}_\mu. \tag{38}$$

(The factor $\frac{1}{2}$ is introduced for later convenience.) In the same way as for Eq. (33) we find

$$k_2 = h_{\mu\nu}\hat{N}^\mu\hat{N}^\nu, \qquad h_{\lambda\nu}\hat{T}^\lambda\hat{N}^\nu = 0. \tag{39}$$

Let us now introduce the whole tetrad basis $\{\hat{T}^\mu, \hat{N}^\mu, \hat{U}^\mu, \hat{V}^\mu\}$; recall $\hat{T}^\mu$ is normal to Σ, $\hat{N}^\mu$ is in Σ but normal to $\partial\Sigma$, and $\hat{U}^\mu, \hat{V}^\mu$ lie in $\partial\Sigma$. The projector onto $\partial\Sigma$ is defined

$$P^{\mu\nu} \equiv (\hat{U}\otimes\hat{U} + \hat{V}\otimes\hat{V})^{\mu\nu} = \hat{U}^\mu\hat{U}^\nu + \hat{V}^\mu\hat{V}^\nu. \tag{40}$$

Similarly

$$g^{\mu\nu} = -\hat{T}^\mu\hat{T}^\nu + \hat{N}^\mu\hat{N}^\nu + P^{\mu\nu}. \tag{41}$$

Now tangent vectors in $\partial\Sigma$ are contained in $\text{span}\{\hat{U}^\mu, \hat{V}^\mu\}$ and since the coordinates of $\partial\Sigma$ are preserved under the diffeomorphism, δU^μ and δV^μ must also be contained in $\text{span}\{\hat{U}^\mu, \hat{V}^\mu\}$. By the same reasoning, $\delta P^{\mu\nu} \in \text{span}\{\hat{U}\otimes\hat{U}, \hat{U}\otimes\hat{V}, \hat{V}\otimes\hat{U}, \hat{V}\otimes\hat{V}\}$.

Let us now use the tetrad decomposition for $\hat{N}^\mu$ to find

$$\begin{aligned} \delta\hat{N}^\mu &= \delta(g^{\mu\nu}\hat{N}_\nu) \\ &= -h^{\mu\nu}\hat{N}_\nu + \frac{1}{2}k_2\hat{N}^\mu \\ &= -g^{\mu\lambda}h_{\lambda\nu}\hat{N}^\nu + \frac{1}{2}k_2\hat{N}^\mu \\ &= -g^{\mu\lambda}(-\delta(\hat{T}_\lambda\hat{T}_\nu) + \delta(\hat{N}_\lambda\hat{N}_\nu) + \delta P_{\lambda\nu})\hat{N}^\nu + \frac{1}{2}k_2\hat{N}^\mu \\ &= -\frac{1}{2}k_2\hat{N}^\mu - g^{\mu\lambda}\delta P_{\lambda\nu}\hat{N}^\nu \\ &= -\frac{1}{2}k_2\hat{N}^\mu. \end{aligned} \tag{42}$$

Note in the fifth line we use $\delta P_{\lambda\nu}\hat{N}^\nu = 0$ since $\delta P_{\mu\nu} \in \text{span}\{\hat{U}\otimes\hat{U}, \hat{U}\otimes\hat{V}, \hat{V}\otimes\hat{U}, \hat{V}\otimes\hat{V}\}$.

Further, since $\delta U^\mu, \delta V^\mu \in \text{span}\{\hat{U}^\mu, \hat{V}^\mu\}$, we may explicitly write them as

$$\delta\hat{U}^\mu = -\frac{1}{2}k_3\hat{U}^\mu - \frac{1}{2}k_4\hat{V}^\mu, \;\; \delta\hat{V}^\mu = -\frac{1}{2}k_5\hat{U}^\mu - \frac{1}{2}k_6\hat{V}^\mu. \tag{43}$$

By considering $\delta(g_{\mu\nu}\hat{U}^\mu\hat{U}^\nu) = 0$, $\delta(g_{\mu\nu}\hat{U}^\mu\hat{V}^\nu) = 0$ and $\delta(g_{\mu\nu}\hat{V}^\mu\hat{V}^\nu) = 0$, it is easy to show that

$$\begin{aligned} k_3 &= h_{\mu\nu}\hat{U}^\mu\hat{U}^\nu \\ k_6 &= h_{\mu\nu}\hat{V}^\mu\hat{V}^\nu \\ k_4 + k_5 &= 2h_{\mu\nu}\hat{U}^\mu\hat{V}^\nu. \end{aligned} \tag{44}$$

Then by considering $\delta(\hat{U}_\mu\hat{U}^\mu) = 0$, $\delta(\hat{U}_\mu\hat{V}^\mu) = 0$, $\delta(\hat{V}_\mu\hat{V}^\mu) = 0$ and $\delta(\hat{V}_\mu\hat{U}^\mu) = 0$, one finds

$$\delta\hat{U}_\mu = \frac{1}{2}k_3\hat{U}_\mu + \frac{1}{2}k_5\hat{V}_\mu, \quad \delta\hat{V}_\mu = \frac{1}{2}k_4\hat{U}_\mu + \frac{1}{2}k_6\hat{V}_\mu. \tag{45}$$

Hence, $\delta P^{\mu\nu}$ may be explicitly computed to be

$$\begin{aligned} \delta P^{\mu\nu} &= -k_3\hat{U}^\mu\hat{U}^\nu - k_6\hat{V}^\mu\hat{V}^\nu \\ &\quad -\frac{1}{2}(k_4+k_5)(\hat{U}^\mu\hat{V}^\nu + \hat{U}^\nu\hat{V}^\mu) \\ &= -\frac{1}{2}(k_3+k_6)(\hat{U}^\mu\hat{U}^\nu + \hat{V}^\mu\hat{V}^\nu) \\ &\quad -\frac{1}{2}(k_3-k_6)(\hat{U}^\mu\hat{U}^\nu - \hat{V}^\mu\hat{V}^\nu) \\ &\quad -\frac{1}{2}(k_4+k_5)(\hat{U}^\mu\hat{V}^\nu + \hat{U}^\nu\hat{V}^\mu) \\ &= -\frac{1}{2}(k_3+k_6)P^{\mu\nu} - \frac{1}{2}(k_3-k_6)(\hat{U}^\mu\hat{U}^\nu - \hat{V}^\mu\hat{V}^\nu) \\ &\quad -\frac{1}{2}(k_4+k_5)(\hat{U}^\mu\hat{V}^\nu + \hat{U}^\nu\hat{V}^\mu), \end{aligned} \tag{46}$$

Similarly,

$$\begin{aligned}\delta P_{\mu\nu} &= k_3\hat{U}_\mu\hat{U}_\nu + k_6\hat{V}_\mu\hat{V}_\nu \\ &+\frac{1}{2}(k_4+k_5)(\hat{U}_\mu\hat{V}_\nu+\hat{U}_\nu\hat{V}_\mu) \\ &= \frac{1}{2}(k_3+k_6)P_{\mu\nu}+\frac{1}{2}(k_3-k_6)(\hat{U}_\mu\hat{U}_\nu-\hat{V}_\mu\hat{V}_\nu) \\ &+\frac{1}{2}(k_4+k_5)(\hat{U}_\mu\hat{V}_\nu+\hat{U}_\nu\hat{V}_\mu). \end{aligned} \quad (47)$$

So the key diffeomorphic conditions may be summarized as

$$\begin{aligned} &\delta K^\mu = 0, & &\delta K_\mu = h_{\mu\nu}K^\nu = k_1 K_\mu \\ &\delta\hat{T}^\mu = -\frac{1}{2}k_1\hat{T}^\mu, & &\delta\hat{T}_\mu = \frac{1}{2}k_1\hat{T}_\mu \\ &\delta\hat{N}^\mu = -\frac{1}{2}k_2\hat{N}^\mu, & &\delta\hat{N}_\mu = \frac{1}{2}k_2\hat{N}_\mu \\ &\delta\hat{T}^t = -\frac{1}{2}k_1\hat{T}^t, & &\hat{T}^\mu h_{\mu\nu}\hat{N}^\nu = 0 \\ &\delta P^{\mu\nu} = \text{Eq.(46)}, & &\delta P_{\mu\nu} = \text{Eq.(47)}. \end{aligned} \quad (48)$$

where $k_1 = -h_{\mu\nu}\hat{T}^\mu\hat{T}^\nu$ and $k_2 = h_{\mu\nu}\hat{N}^\mu\hat{N}^\nu$.

From Eqs. (33), (39) and (44), we know that in the tetrad basis that

$$h_{\hat{\mu}\hat{\nu}} = \begin{pmatrix} -k_1 & 0 & 0 & 0 \\ 0 & k_2 & 0 & 0 \\ 0 & 0 & k_3 & \frac{k_4+k_5}{2} \\ 0 & 0 & \frac{k_4+k_5}{2} & k_6 \end{pmatrix}, \quad (49)$$

i.e., $h_{\hat{T}\hat{T}} = -k_1, h_{\hat{N}\hat{N}} = k_2$ etc.. So $k_2, k_3, \ldots, k_6$ are independent functions from each other.

Reduction to the first law

Since $K^\mu\hat{N}_\mu = 0$, then $(K^\mu\hat{N}_\mu)_{;\nu} = 0$, and so

$$K^\mu{}_{;\nu}\hat{N}_\mu = -K^\mu\hat{N}_{\mu;\nu}. \quad (50)$$

We then consider the expansion of null normal congruences on the inner boundary which may be written as

$$\begin{aligned}\theta^{(l)} &= P^{\mu\nu}l_{\mu;\nu} \\ &= P^{\mu\nu}(\hat{T}_\mu+\hat{N}_\mu)_{;\nu} \\ &= P^{\mu\nu}(\hat{T}^tK_\mu)_{;\nu}+P^{\mu\nu}\hat{N}_{\mu;\nu} \\ &= \hat{T}^tP^{\mu\nu}K_{\mu;\nu}+P^{\mu\nu}K_\mu(\hat{T}^t)_{;\nu}+P^{\mu\nu}\hat{N}_{\mu;\nu} \\ &= P^{\mu\nu}\hat{N}_{\mu;\nu} \\ &= (g^{\mu\nu}+\hat{T}^\mu\hat{T}^\nu-\hat{N}^\mu\hat{N}^\nu)\hat{N}_{\mu;\nu} \\ &= \hat{N}^\mu{}_{;\mu}-K_{\mu;\nu}\hat{T}^\nu\hat{N}^\mu\hat{T}^t \\ &= \hat{N}^\mu{}_{;\mu}-\kappa\hat{T}^t \\ &= \frac{1}{\sqrt{-g}}(\sqrt{-g}\hat{N}^\mu)_{,\mu}-\kappa\hat{T}^t \end{aligned} \quad (51)$$

where $l_\mu = \hat{T}_\mu+\hat{N}_\mu$ is the outgoing null normal vector of the inner boundary, and we have used Eq. (41) in the fifth line and Eq. (50) in the sixth line. Using this relation we may express the variation of $\kappa\hat{T}^t$ as

$$\begin{aligned}\delta\hat{T}^t\kappa+\hat{T}^t\delta\kappa &= \delta(\frac{1}{\sqrt{-g}})(\sqrt{-g}\hat{N}^\mu)_{,\mu}+\frac{1}{\sqrt{-g}}(\delta\sqrt{-g}\hat{N}^\mu)_{,\mu}+\frac{1}{\sqrt{-g}}(\sqrt{-g}\delta\hat{N}^\mu)_{,\mu}-\delta\theta^{(l)} \\ -\frac{1}{2}k_1\hat{T}^t\kappa+\hat{T}^t\delta\kappa &= -\frac{1}{2}g^{\tau\nu}\delta g_{\tau\nu}\frac{1}{\sqrt{-g}}(\sqrt{-g}\hat{N}^\mu)_{,\mu}+\frac{1}{\sqrt{-g}}(\frac{1}{2}\sqrt{-g}g^{\tau\nu}\delta g_{\tau\nu}\hat{N}^\mu)_{,\mu}+(\delta\hat{N}^\mu)_{;\mu}-\delta\theta^{(l)} \\ &= -\frac{1}{2}g^{\tau\nu}\delta g_{\tau\nu}\frac{1}{\sqrt{-g}}(\sqrt{-g}\hat{N}^\mu)_{,\mu}+\frac{1}{2}g^{\tau\nu}\delta g_{\tau\nu}\frac{1}{\sqrt{-g}}(\sqrt{-g}\hat{N}^\mu)_{,\mu}+\frac{1}{2}(g^{\tau\nu}\delta g_{\tau\nu})_{,\mu}\hat{N}^\mu+(\delta\hat{N}^\mu)_{;\mu}-\delta\theta^{(l)} \\ &= \frac{1}{2}(h_\nu{}^\nu)_{,\mu}\hat{N}^\mu+(\delta\hat{N}^\mu)_{;\mu}-\delta\theta^{(l)}, \end{aligned} \quad (52)$$

where $\delta\sqrt{-g} = \frac{1}{2}\sqrt{-g}g^{\mu\nu}\delta g_{\mu\nu}$. Hence, the first term in Eq. (28) may be written as

$$\frac{1}{2}(h_\nu{}^\nu)_{;\mu}\hat{N}^\mu = -\frac{1}{2}k_1\hat{T}^t\kappa+\hat{T}^t\delta\kappa-(\delta\hat{N}^\mu)_{;\mu}+\delta\theta^{(l)} \quad (53)$$

The second term ${h_\mu}^{\nu;\mu}\hat{N}_\nu$ in Eq.(28) can then be expressed as

$$\begin{aligned}
\frac{1}{2}{h_\mu}^{\nu;\mu}\hat{N}_\nu &= \frac{1}{2}({h_\mu}^\nu\hat{N}_\nu)^{;\mu} - \frac{1}{2}{h_\mu}^\nu{\hat{N}_\nu}{}^{;\mu} = \frac{1}{2}(h_{\mu\nu}\hat{N}^\nu)^{;\mu} - \frac{1}{2}h^{\mu\nu}\hat{N}_{\nu;\mu} = \frac{1}{2}(\delta g_{\mu\nu}\hat{N}^\nu)^{;\mu} + \frac{1}{2}\delta g^{\mu\nu}\hat{N}_{\mu;\nu} \\
&= \frac{1}{2}\big(\delta(g_{\mu\nu}\hat{N}^\nu) - g_{\mu\nu}\delta\hat{N}^\nu\big)^{;\mu} + \frac{1}{2}\delta(-\hat{T}^\mu\hat{T}^\nu + \hat{N}^\mu\hat{N}^\nu + P^{\mu\nu})\hat{N}_{\mu;\nu} \\
&= \frac{1}{2}(\delta\hat{N}_\mu - \delta\hat{N}^\nu g_{\mu\nu})^{;\mu} + \frac{1}{2}(k_1\hat{T}^\mu\hat{T}^\nu - k_2\hat{N}^\mu\hat{N}^\nu + \delta P^{\mu\nu})\hat{N}_{\mu;\nu} \\
&= \frac{1}{2}(\frac{1}{2}k_2\hat{N}_\mu)^{;\mu} - \frac{1}{2}(\delta\hat{N}^\mu)_{;\mu} + \frac{1}{2}k_1\hat{T}^\mu\hat{T}^\nu\hat{N}_{\mu;\nu} + \frac{1}{2}\delta P^{\mu\nu}\hat{N}_{\mu;\nu} \\
&= \frac{1}{2}(\frac{1}{2}k_2\hat{N}^\mu)_{;\mu} - \frac{1}{2}(\delta\hat{N}^\mu)_{;\mu} - \frac{1}{2}k_1\hat{T}^t\hat{T}^\nu\hat{N}_\mu {K^\mu}_{;\nu} + \frac{1}{2}\delta P^{\mu\nu}\hat{N}_{\mu;\nu} \\
&= -(\delta\hat{N}^\mu)_{;\mu} - \frac{1}{2}k_1\hat{T}^t\kappa + \frac{1}{2}\delta P^{\mu\nu}\hat{N}_{\mu;\nu}, \qquad (54)
\end{aligned}$$

where we have used Eq. (50) in the fifth line, and Eqs. (7) and (48) in the last step.

Next, consider the final term $\frac{1}{2}\delta P^{\mu\nu}\hat{N}_{\mu;\nu}$ in Eq. (54), using Eq. (46) we have

$$\begin{aligned}
\frac{1}{2}\delta P^{\mu\nu}\hat{N}_{\mu;\nu} &= -\frac{1}{4}\Big((k_3+k_6)P^{\mu\nu} + (k_3-k_6)(\hat{U}^\mu\hat{U}^\nu - \hat{V}^\mu\hat{V}^\nu) + (k_4+k_5)(\hat{U}^\mu\hat{V}^\nu + \hat{U}^\nu\hat{V}^\mu)\Big)\hat{N}_{\mu;\nu} \\
&= -\frac{1}{4}\Big((k_3+k_6)P^{\mu\nu} + (k_3-k_6)(\hat{U}^\mu\hat{U}^\nu - \hat{V}^\mu\hat{V}^\nu) + (k_4+k_5)(\hat{U}^\mu\hat{V}^\nu + \hat{U}^\nu\hat{V}^\mu)\Big)(l_\mu - \hat{T}_\mu)_{;\nu} \\
&= -\frac{1}{4}\Big((k_3+k_6)P^{\mu\nu} + (k_3-k_6)(\hat{U}^\mu\hat{U}^\nu - \hat{V}^\mu\hat{V}^\nu) + (k_4+k_5)(\hat{U}^\mu\hat{V}^\nu + \hat{U}^\nu\hat{V}^\mu)\Big)(l_\mu - \hat{T}^t K_\mu)_{;\nu} \\
&= -\frac{1}{4}(k_3+k_6)\theta^{(l)} - \frac{1}{4}(k_3-k_6)\sigma_+^{(l)} - \frac{1}{4}(k_4+k_5)\sigma_\times^{(l)}, \qquad (55)
\end{aligned}$$

where $\sigma_+^{(l)}, \sigma_\times^{(l)}$ are the shears of l^μ defined by [10]

$$\sigma_+^{(l)} = (\hat{U}^\mu\hat{U}^\nu - \hat{V}^\mu\hat{V}^\nu)l_{\mu;\nu} \qquad \sigma_\times^{(l)} = (\hat{U}^\mu\hat{V}^\nu + \hat{U}^\nu\hat{V}^\mu)l_{\mu;\nu} \ . \qquad (56)$$

Therefore,

$$\frac{1}{2}{h_\mu}^{\nu;\mu}\hat{N}_\nu = -(\delta\hat{N}^\mu)_{;\mu} - \frac{1}{2}k_1\hat{T}^t\kappa - \frac{1}{4}(k_3+k_6)\theta^{(l)} - \frac{1}{4}(k_3-k_6)\sigma_+^{(l)} - \frac{1}{4}(k_4+k_5)\sigma_\times^{(l)}. \qquad (57)$$

Finally, substituting Eq.(53) and Eq.(57) into Eq.(28), we find

$$\begin{aligned}
\delta M = & -\frac{1}{8\pi}\int_{\partial\Sigma_{\rm in}}\left(\delta\kappa + \frac{1}{\hat{T}^t}\Big(\delta\theta^{(l)} + \frac{1}{4}(k_3+k_6)\theta^{(l)} + \frac{1}{4}(k_3-k_6)\sigma_+^{(l)} + \frac{1}{4}(k_4+k_5)\sigma_\times^{(l)}\Big)\right) dA \\
& + \frac{1}{8\pi}\int_{\partial\Sigma_{\rm in}}\delta\kappa \, dA + \frac{1}{8\pi}\int_{\partial\Sigma_{\rm in}}\kappa \, \delta(dA) \ . \qquad (58)
\end{aligned}$$

Or in summary,

$$\boxed{\delta M = -\frac{1}{8\pi}\int_{\partial\Sigma_{\rm in}}\left(\delta\theta^{(l)} + \frac{1}{4}(k_3+k_6)\theta^{(l)} + \frac{1}{4}(k_3-k_6)\sigma_+^{(l)} + \frac{1}{4}(k_4+k_5)\sigma_\times^{(l)}\right)\mathcal{N} \, dA + \frac{1}{8\pi}\int_{\partial\Sigma_{\rm in}}\kappa \, \delta(dA).} \qquad (59)$$

It is worth noting that

$$\boxed{\delta\theta^{(l)} = -\frac{k_2}{2}\theta^{(l)} + \frac{1}{2}(k_3+k_6)_{;\rho}\hat{N}^\rho} \qquad (60)$$

which separately depends only on k_2, k_3, k_6, and we will prove it next.

Since $P^{\mu\nu}\hat{T}_{\mu;\nu} = P^{\mu\nu}(K_\mu\hat{T}^t)_{;\nu} = P^{\mu\nu}K_{\mu;\nu}\hat{T}^t + P^{\mu\nu}K_\mu(\hat{T}^t)_{;\nu} = 0$, $\theta^{(l)}$ can be simplified as

$$\theta^{(l)} = P^{\mu\nu}l_{\mu;\nu} = P^{\mu\nu}(\hat{T}_{\mu;\nu} + \hat{N}_{\mu;\nu}) = P^{\mu\nu}\hat{N}_{\mu;\nu}. \qquad (61)$$

Thus the variation of $\theta^{(l)}$ is

$$\begin{aligned}
\delta\theta^{(l)} &= \delta P^{\mu\nu}\hat{N}_{\mu;\nu} + P^{\mu\nu}\delta(\hat{N}_{\mu;\nu}) \\
&= \delta P^{\mu\nu}\hat{N}_{\mu;\nu} + P^{\mu\nu}(\delta\hat{N}_{\mu,\nu} - \delta\Gamma^{\lambda}_{\mu\nu}\hat{N}_{\lambda} - \Gamma^{\lambda}_{\mu\nu}\delta\hat{N}_{\lambda}) \\
&= \delta P^{\mu\nu}\hat{N}_{\mu;\nu} + P^{\mu\nu}\Big((\delta\hat{N}_{\mu})_{;\nu} - \delta\Gamma^{\lambda}_{\mu\nu}\hat{N}_{\lambda}\Big) \\
&= \delta P^{\mu\nu}\hat{N}_{\mu;\nu} + P^{\mu\nu}\Big((\frac{k_2}{2}\hat{N}_{\mu})_{;\nu} - \frac{1}{2}g^{\lambda\rho}(h_{\mu\rho;\nu} + h_{\nu\rho;\mu} - h_{\mu\nu;\rho})\hat{N}_{\lambda}\Big) \\
&= \delta P^{\mu\nu}\hat{N}_{\mu;\nu} + P^{\mu\nu}(\frac{k_2}{2}\hat{N}_{\mu})_{;\nu} - P^{\mu\nu}\frac{1}{2}(h_{\mu\rho;\nu} + h_{\nu\rho;\mu} - h_{\mu\nu;\rho})\hat{N}^{\rho} \\
&= \delta P^{\mu\nu}\hat{N}_{\mu;\nu} + P^{\mu\nu}(\frac{k_2}{2}\hat{N}_{\mu})_{;\nu} - P^{\mu\nu}h_{\mu\rho;\nu}\hat{N}^{\rho} + \frac{1}{2}P^{\mu\nu}h_{\mu\nu;\rho}\hat{N}^{\rho} \\
&= \delta P^{\mu\nu}\hat{N}_{\mu;\nu} + P^{\mu\nu}(\frac{k_2}{2}\hat{N}_{\mu})_{;\nu} - (\hat{N}^{\rho}h_{\mu\rho})_{;\nu}P^{\mu\nu} + \hat{N}^{\rho}{}_{;\nu}P^{\mu\nu}h_{\mu\rho} + \frac{1}{2}(P^{\mu\nu}h_{\mu\nu})_{;\rho}\hat{N}^{\rho} - \frac{1}{2}P^{\mu\nu}{}_{;\rho}\hat{N}^{\rho}h_{\mu\nu} \\
&= \delta P^{\mu\nu}\hat{N}_{\mu;\nu} + P^{\mu\nu}(\frac{k_2}{2}\hat{N}_{\mu})_{;\nu} - (k_2\hat{N}_{\mu})_{;\nu}P^{\mu\nu} + \hat{N}^{\rho}{}_{;\nu}(\delta(P^{\mu\nu}g_{\mu\rho}) - \delta P^{\mu\nu}g_{\mu\rho}) + \frac{1}{2}(k_3 + k_6)_{;\rho}\hat{N}^{\rho} \\
&= \delta P^{\mu\nu}\hat{N}_{\mu;\nu} - P^{\mu\nu}(\frac{k_2}{2}\hat{N}_{\mu})_{;\nu} - \delta P^{\mu\nu}\hat{N}_{\mu;\nu} + \frac{1}{2}(k_3 + k_6)_{;\rho}\hat{N}^{\rho} \\
&= -\frac{k_2}{2}P^{\mu\nu}\hat{N}_{\mu;\nu} + \frac{1}{2}(k_3 + k_6)_{;\rho}\hat{N}^{\rho} \\
&= -\frac{k_2}{2}\theta^{(l)} + \frac{1}{2}(k_3 + k_6)_{;\rho}\hat{N}^{\rho},
\end{aligned} \tag{62}$$

where we have used Eq. (18) in the fourth line and $P^{\mu\nu}{}_{;\rho}\hat{N}^{\rho}h_{\mu\nu} = 0$ in the seventh line.

This completes the proof of Eq. (60). □

Vanishing extrinsic curvature tensor

Our analysis has been predicated on static screens. However, there is another way to define screens, so their normal direction remains parallel to the proper acceleration of a family of locally coincident timelike observers [12]. These observers are constrained to have constant 4-acceleration along with a number of other technical assumptions [12]. A first law is then obtained for these surfaces provided they additionally have a vanishing extrinsic curvature tensor $K_{\mu\nu} = 0$ [12]. The first law obtained is of a form with energy and temperature measured locally instead of at spatial infinity, which for asymptotically-flat spacetimes are unambiguous. Finally, we note that there is no easy way in this other formalism [12] to investigate stretched horizons.

In our setting with zero shift vector $\beta^{\mu} = 0$, so $\hat{T}^{\mu} = \hat{T}^{t}K^{\mu}$, and our hypersurfaces Σ are orthogonal to $\hat{T}^{\mu}$, we find that $K_{\mu\nu} = 0$ implies a vanishing expansion $\theta^{(l)} = 0$. Thus, for our setting, the formalism of Ref. 12 only yields a first law on horizons.

To see that this is the case, recall that the extrinsic curvature tensor of our inner boundary equals [3]

$$K_{\mu\nu} \equiv \hat{N}_{(\lambda;\rho)}P^{\lambda}{}_{\mu}P^{\rho}{}_{\nu}. \tag{63}$$

Taking the trace of this yields the extrinsic curvature scalar as $K = P^{\mu\nu}\hat{N}_{\mu;\nu} = \theta^{(l)}$, where in the final step we use Eq. (61). Thus, for our setting, the first law of Ref. 12 appears to occur at the horizon; a result which is naively consistent with the classic 1973 result.

Let us now consider a construction for a screen surrounding a gravitating body as proposed by Ref. 12: Construct a screen using a family of stationary timelike observers at fixed radius around a Schwarzschild black hole. It is easy to calculate the extrinsic curvature tensor for the screen and see, as noted above, that this curvature vanishes only on the horizon. Hence the screen is on the horizon and the observers are null instead of timelike observers. Next drop in a spherical shell of matter. As the shell passes the screen of observers, the horizon (where $\theta^{(l)} = 0$) discontinuously jumps, the surface gravity of the new horizon changes and the original screen of observers fall into the black hole. We must then conclude either that the construction using the methods of Ref. 12 of a screen surrounding the black hole is simply impossible (because the observers are not timelike), or it fails to continue to hold under perturbation.

Thus, although Ref. 12 purports to describe a dynamical first law for ordinary surfaces its conditions are either in general impossible to satisfy or are generally *not* preserved under perturbation.

Local temperature in emergent gravity

We focus here on the temperature defined in the original paper on emergent gravity [4] which is used there in

a heuristic derivation of the Einstein field equations. In Fig. 2 we show a schematic of the hypersurface considered there. $\partial\Sigma_{\rm HS}$ denotes the holographic screen (ordinary surfaces of constant Newtonian potential ϕ) which now is the outer boundary of the spacelike hypersurface $\Sigma_{\rm EG}$ under study, and $\hat{N}^\mu$ is the unit normal vector of the holographic screen.

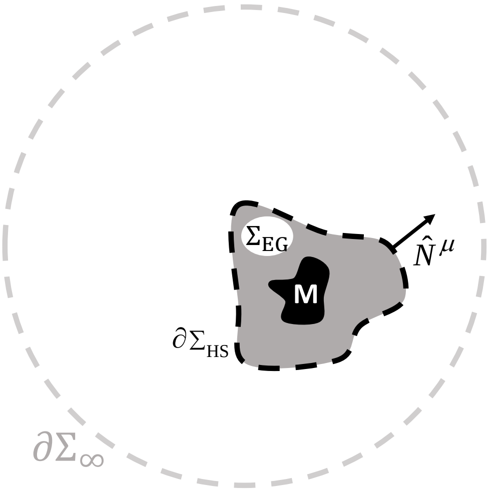

FIG. 2: Schematic of the spacelike three-dimensional hypersurface $\Sigma_{\rm EG}$ used in Ref. 4 which has the mass under study embedded within it. As can be seen, the 2-surface corresponding to the holographic screen $\partial\Sigma_{\rm HS}$ is now the *outer* boundary to $\Sigma_{\rm EG}$ (compare to Fig. 1); and Ref. 4 defines $\partial\Sigma_{\rm HS}$ as ordinary surfaces of constant Newtonian potential ϕ. (For context, we show spatial infinity as $\partial\Sigma_\infty$ in grey, though it plays no roll in this section.)

The 'local' temperature of the holographic screen (as measured at spatial infinity) used in Ref. 4 is defined as

$$T \equiv \frac{1}{2\pi} e^\phi \, \phi_{;\mu} \hat{N}^\mu, \tag{64}$$

where ϕ is the generalized Newtonian potential, given by $\phi = \frac{1}{2}\ln(-K^\mu K_\mu) = \ln\mathcal{N}$, recalling that $K^\mu K_\mu = -\mathcal{N}^2$. It is now an easy matter to check that

$$\begin{aligned} T &\equiv \frac{1}{2\pi} e^\phi \, \phi_{;\mu}\hat{N}^\mu = \frac{1}{2\pi}\mathcal{N}_{;\mu}\hat{N}^\mu = \frac{1}{2\pi}\frac{1}{2\mathcal{N}}(\mathcal{N}^2)_{;\mu}\hat{N}^\mu \\ &= -\frac{1}{2\pi}K_{\nu;\mu}\frac{1}{\mathcal{N}}\,K^\nu\hat{N}^\mu = \frac{1}{2\pi}K_{\mu;\nu}\frac{1}{\mathcal{N}}\,K^\nu\hat{N}^\mu \\ &= \frac{1}{2\pi}K_{\mu;\nu}\hat{T}^\nu\hat{N}^\mu. \end{aligned} \tag{65}$$

In summary, recall the definition of κ in Eq. (7), yielding

$$\boxed{T \equiv \frac{\kappa}{2\pi}.} \tag{66}$$

For reference, the Unruh temperature associated with a stationary observer is just the *magnitude* of the observer's proper acceleration a^μ over 2π. As their 4-velocity is given by $\hat{T}^\mu$ we easily find

$$a^\mu \equiv \hat{T}^\mu{}_{;\nu}\hat{T}^\nu = \phi^{;\mu}, \tag{67}$$

since $\hat{T}^\mu = \hat{T}^t K^\mu = K^\mu/\mathcal{N} = e^{-\phi}K^\mu$. Thus a^μ is perpendicular to surfaces of constant ϕ. When Verlinde's temperature is measured locally (instead of referenced to spatial infinity) it is $T_{\rm local} = \frac{1}{2\pi}\phi^{;\mu}\hat{N}_\mu$. For this to equal the Unruh temperature at the same point, the local unit normal $\hat{N}^\mu$ to the screen must be aligned with the proper acceleration a^μ of our stationary observer there. Therefore, it trivially follows that only for surfaces of constant Newtonian potential ϕ would the holographic screens be in thermal equilibrium with stationary physical surfaces of the same shape, size and location. Hence,

$$\boxed{\text{Thermodynamic equilibrium} \quad \Rightarrow \quad \hat{N}^\mu \parallel \phi^{;\mu}} \tag{68}$$

Finally, we show that for surfaces of constant ϕ, we have $\delta\phi = k_1/2$. Indeed, since $\hat{T}^t = 1/\mathcal{N} = e^{-\phi}$, we have

$$\boxed{\delta\phi = -\frac{1}{\hat{T}^t}\delta\hat{T}^t = \frac{1}{2}k_1,} \tag{69}$$

where in the last step we have used Eqs. (36) and (37).

Acknowledgements. Z-WW acknowledges the support from Jilin University and China Scholarships Council. Z-WW is grateful to Tang Aoqing Honors Program in Science.

* Electronic address: phyjasonwang@gmail.com; Electronic address: sam.braunstein@york.ac.uk

[1] Bardeen, J. M., Carter, B. & Hawking, S. W. The four laws of black hole mechanics. *Communications in Mathematical Physics* **31**, 161–170 (1973).

[2] De, S. V. & Zhang, Z. Black hole physics. (Springer Science & Business Media, 1992), pp55-79.

[3] Carroll, S. M. An Introduction to General Relativity Spacetime and Geometry (Addison Wesley, San Francisco, 2004). p251, p450, p456.

[4] Verlinde, E. On the Origin of Gravity and the Laws of Newton. *Journal of High Energy Physics* **2011**, 29 (2011).

[5] Gourgoulhon, E. 3+ 1 Formalism in General Relativity: Bases of Numerical Relativity (Springer Science & Business Media, 2012). pp79-84.

[6] Bertschinger, E. Symmetry transformations, the Einstein-Hilbert action and gauge invariance. *Massachusetts Institute of Technology, Department of Physics* (2002).

[7] Palatini, A. Deduzione invariantiva delle equazioni gravitazionali dal principio di Hamilton. *Rendiconti del Circolo Matematico di Palermo (1884-1940)* **43**, 203–212 (1919).

[8] Poisson, E. A relativist's toolkit: the mathematics of black-hole mechanics. (Cambridge university press, 2004), p15, pp211-212.

[9] Wald, R. M. General relativity. (University of Chicago press, 1984), p289, p336, p453, p463.

[10] Hawking, S. W. & Ellis, G. F. R. The large scale structure of space-time. (Cambridge university press, 1973), pp82-83, p334,.

[11] Chen, Y.-X. & Li, J.-L. First law of thermodynamics on holographic screens in entropic force frame. *Physics Letters B* **700**, 380–384 (2011).

[12] Piazza, F. Gauss-Codazzi thermodynamics on the timelike screen. *Physical Review D* **82**, 084004 (2010).
